# The Compromised Mucosal Immune System of β7 Integrin-Deficient Mice Has Only Minor Effects on the Fecal Microbiota in Homeostasis

**DOI:** 10.3389/fmicb.2019.02284

**Published:** 2019-10-04

**Authors:** Anshu Babbar, Thomas C. A. Hitch, Oliver Pabst, Thomas Clavel, Jessica Hübel, Sreepradha Eswaran, Norbert Wagner, Angela Schippers

**Affiliations:** ^1^Department of Pediatrics, Faculty of Medicine, RWTH Aachen University, Aachen, Germany; ^2^Functional Microbiome Research Group, Institute of Medical Microbiology, Faculty of Medicine, RWTH Aachen University, Aachen, Germany; ^3^Institute of Molecular Medicine, Faculty of Medicine, RWTH Aachen University, Aachen, Germany

**Keywords:** gut microbiota, mucosal immune system, β7 integrin, 16S rRNA amplicon sequencing, *Muribaculaceae*

## Abstract

The gastrointestinal tract is an ideal habitat for diverse bacterial species that reside in a homeostatic balance with local tissue and significantly contribute to host health. Negative shifts in gut microbiota profiles, also known as dysbiosis, may be implicated in the development of chronic disorders such as inflammatory bowel diseases (IBD). Adhesion molecule-dependent recruitment of immune cells to the gut is an important step in IBD pathogenesis. The adhesion molecule β7 integrin contributes to the development of the gut-associated lymphoid tissue (GALT), intestinal immune cell homing, and immune responses and is known to promote intestinal inflammation. Although many studies underlined the role of the gut microbiota in shaping the mucosal immune system, studies on the influence of the host immune system on the microbiota are rare, especially in homeostasis. We addressed this question via comparative 16S rRNA gene amplicon analysis of fecal microbial communities from wild-type and β7 integrin-deficient mice, the latter being characterized by a compromised GALT. Besides subtle changes in relative abundances of *Muribaculaceae* spp. and unknown members of the families *Ruminococcaceae* and *Lachnospiraceae*, there was altogether no major difference in microbiota profiles in β7 integrin-deficient mice vs. wild-type littermates. This indicates that, in conditions of homeostasis, there is only a minor influence of the host immune system on the fecal microbiota in our mouse model, stressing the potential importance of pathological factors for dysbiosis development.

## Introduction

The gastrointestinal tract of humans and mice is inhabited by approximately 100 trillion microbes including bacteria, viruses, fungi and protozoa that altogether constitute the “gut microbiota” (Honda and Littman, 2012). During homeostasis, the gut microbiota confers benefits to the host, including colonization resistance against pathogens, production of vitamins and other nutrients, and shaping the development of immune responses (Hill and Artis, 2010; Renz et al., 2011; Sonnenberg and Artis, 2012; Nishida et al., 2018). Alterations of the structure or functions of the gut microbiota, also referred to as dysbiosis, can be associated with chronic diseases such as IBD (Henson and Phalak, 2017; Lloyd-Price et al., 2019). Moreover, it has recently been shown that fecal microbiota transplantation is capable of therapeutically controlling intestinal experimental colitis and initiates restoration of intestinal homeostasis (Burrello et al., 2018).

Defending against pathogens whilst allowing tolerance is a prerequisite for the maintenance of homeostasis. For this purpose, the gut associated lymphoid tissues [(GALT) comprising organized lymphatic tissues including Peyer’s patches (PPs)], gut draining mesenteric lymph nodes (MLNs), and immune cells residing in the gut epithelium and lamina propria (LP), act as active sites for lymphocyte trafficking and retention (Brandtzaeg et al., 2007). Migration of immune cells is controlled by the expression of chemokines and adhesion molecules in the target tissue, which recruits specific immune cells bearing the appropriate receptors (Butcher, 1991; von Andrian and Mackay, 2000; Ley et al., 2007; Gorfu et al., 2009). The efficient homing and retention of immune cells in the GALT is dependent on β7 integrin, which forms dimers with either the α4 or αE (CD103) integrin subunit (Kunkel and Butcher, 2002; Gorfu et al., 2009). α4β7 integrin is expressed on a multitude of adoptive and innate immune cells. It directs their migration into the GALT mainly via interactions with its endothelial ligand mucosal addressin cell adhesion molecule-1 (MAdCAM-1), whereas αEβ7 integrin facilitates the retention of lymphocytes in the gut epithelium via binding to E-Cadherin (Wagner et al., 1996; Habtezion et al., 2016). β7 integrin/MAdCAM-1-mediated homing of immune cells contributes to the development of immunogenic and tolerogenic immune responses in the gastrointestinal tract and has been shown to promote inflammatory bowel diseases (IBD) (Sydora et al., 2002; Bamias et al., 2013; Singh et al., 2016). The GALT of β7 integrin-deficient mice is severely compromised, including hypocellular PP with strongly reduced size, a reduced number of IgA-secreting plasma cells, a diminished number of intestinal intraepithelial lymphocytes, and decreased frequencies of intraepithelial CD11c^+^ cells including CD103^+^ dendritic cells (cDC) and plasmacytoid dendritic cells (pDC) in comparison to wild-type mice (Wagner et al., 1996; Schippers et al., 2012; Clahsen et al., 2015).

A humanized antibody (Vedolizumab), which selectively targets α4β7 integrin is used for the treatment of ulcerative colitis and Crohn’s disease. In addition, it has recently been shown that a higher diversity in the microbiota at the outset of IBD treatment predicts a better response to Vedolizumab treatment and patients achieving an early remission exhibited a significantly higher community α-diversity in comparison to non-responders (Ananthakrishnan et al., 2017). However, the role of β7 integrin in the homeostatic relationship between the mucosal immune system of the gut and the microbiota still needs to be investigated.

To explore the effects of a compromised GALT on the gut microbiota in homeostasis, we performed a comparative microbiota analysis in feces from C57BL/6J wild-type (WT) and β7 integrin-deficient mice [B6-(Intβ7)tm (β7^–/–^)]. No major influence of the β7 integrin deficiency and thereby the different immune cell composition of the compromised GALT on the gut microbiota structure was observed, suggesting that, at least in homeostasis, environmental factors rather than immune cell compartments shape the gut microbiota.

## Materials and Methods

### Mice and Sample Collection

All experiments were performed in accordance with the German guidelines for animal housing and husbandry and were approved by the local Institutional Animal Care and Research Advisory Committee and authorized by the regional government authorities for nature, environmental and consumer protection of North Rhine-Westphalia (LANUV – Landesamt für Natur, Umwelt und Verbraucherschutz NRW) Recklinghausen, Germany (approval no. 81-02.04.2017.A429). Mice were kept in specific pathogen-free conditions and used for feces collection at different time points (Supplementary Table S1). The β7 integrin-deficient mouse line C57BL/6-Itgb^tm 1Cgn^/J (β7^–/–^) was originally described in Wagner et al. (1996). All samples were collected from littermate offspring animals from heterozygous breeding pairs [Co-housing of WT (C57BL/6J) and β7^–/–^ mutant] and stored at −20°C until further processing.

### DNA Isolation

Metagenomic DNA was extracted from frozen fecal pellets. Bacteria were disrupted using zirconia beads (Biospec) and a FastPrep 24 device (MP Biomedical, United States) at 6 m/s for 20 sec. Homogenates were then incubated at 80°C for 10 min, centrifuged at 17000 × g for 1 min and supernatant was used for DNA isolation using the QIAamp fast DNA stool kit (Qiagen, Cat. No. 51604) according to the supplier’s instruction.

### 16S rRNA Gene Amplicon Sequencing and Data Analysis

The V4 hypervariable region of the 16S rRNA genes was amplified using primers 515F and 806R (Apprill et al., 2015; Parada et al., 2016). PCR was performed using high fidelity AccuPrime^TM^ *Taq* DNA Polymerase (Invitrogen, Cat. No. 12346086), including 35 cycles with an annealing temperature of 50°C. PCR clean-up and removal of small fragments was done with the Nucleo Spin Gel and PCR Clean-up Kit (Macherey-Nagel, Cat. No. 740609.250). Quantification of extracted PCR products was performed using PicoGreen assay (QuantIT, Thermo Fisher Scientific, Cat. No. P11496). Thereafter, pooling and sequencing of sample-specific libraries were performed by following the Illumina MiSeq protocol. Data processing was done using the IMNGS platform (Lagkouvardos et al., 2016), applying the UPARSE analysis pipeline (Edgar, 2013) with the following settings: Number of allowed mismatches in the barcode: 1, Min fastq quality score for trimming of unpaired reads: 20, Max rate of expected errors in paired sequences: 2, Minimum relative abundance of Operational Taxonomic Units (OTU) cutoff (0-1): 0.25%. The taxonomy of OTUs clustered at 97% sequence identity was determined using SILVA^1^ (Pruesse et al., 2012). The data were submitted to the Sequence Read Archive and are available under the accession number PRJNA514431.

### Statistical Analysis

Statistical analyses were performed using Rhea in the R programming environment (Lagkouvardos et al., 2017). Alpha- and beta-diversity were calculated from normalized data using generalized UniFrac distances in the latter case. Visualization of the multidimensional distance matrix was achieved through either Multi-Dimensional Scaling (MDS) or its Non-metric counterpart (NMDS).

### Flow Cytometry

IEL and LPL fractions of small intestine and colon were prepared and stained as mentioned before (Hadis et al., 2011, *Immunity*). The cells were analyzed on FACS CANTO-II (BD BioSciences). Analysis of major immune cell populations was performed after staining with the following antibodies: CD45 (APC-Cy7), CD19 (eFlour 450), CD3e (PerCP-Cy5.5), CD11c (APC), PDCA-1 (PE), CD8 (Brilliant Violet 510) (all from eBioSciences) and CD4 (APC) and CD11b (AmCyan) (both from BD Biosciences). For ILC analysis cells were stained with biotinylated antibodies to the following common lineage markers: CD3e, CD8, B220, CD11b, CD11c, Gr1, F4/80, CD19, NK1.1 (all from eBioSciences), CD5, TER-119 (both from BioLegend) and streptavidin APC (BD Biosciences) and with RORγt (PE, eBioscience), and CD127 (APC-CY7), T-bet (PE-Cy7) and GATA3 (PB) (all from BioLegend).

## Results and Discussion

The major focus of the present study was to understand the impact of genotype, driving here major changes in the GALT, on the fecal microbiota structure in homeostasis. There have been many reports showing knock-out dependent differences in the gut microbiota of mice (Okubo et al., 2016; de Bruyn et al., 2018; Sun et al., 2019). However, in many cases, confounding factors that might have contributed to the differences observed have been neglected, such as breeders and cage-driven effects (Robertson et al., 2019).

Initially, we performed an immunological analysis of major immune cell populations in the intestines of β7 integrin-deficient (β7^–/–^) mice (10–11 weeks old) and wild-type mice (C57BL/6J) of comparable age to guaranty that the immune status of our mice strains concords with previously published literature. Consistent with previously published data, β7 integrin-deficient mice exhibited reduced frequencies of small intestinal CD19^+^ B cells, CD8^+^ T cells, pDC, and cDC and decreased frequencies of colonic CD19^+^ B cells, CD8^+^ T cells, and pDC in comparison to wild-type mice (Supplementary Figure S1). In addition, the average size of PP in the small intestines from β7 integrin-deficient mice was strongly reduced to approximately 10% of that in wild-type mice (data not shown). With respect to innate lymphoid cells (ILC) a previous study (Kim et al., 2015) showed decreased frequencies of ILC1 and ILC3 in the lamina propria of β7 integrin-deficient mice (Kim et al., 2015). However, we observe only very low numbers and no significant differences in the frequencies of intestinal ILC subsets between wild-type and β7 integrin-deficient mice (Supplementary Figure S2), most probably due to inter-facility variations and the SPF status of our mice.

**FIGURE 1:**
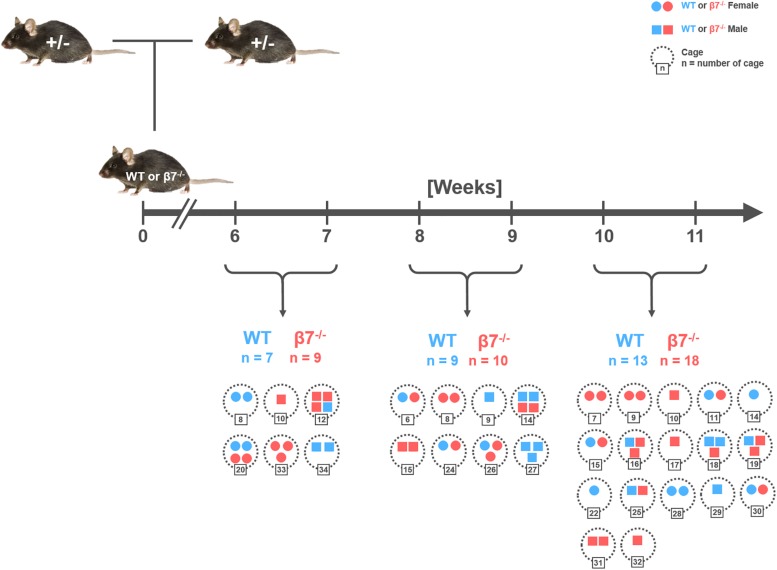
Schematic diagram of the breeding and sampling strategy. Fecal samples were collected from littermates of wild-type (WT; C57BL/6J) and homozygous β7 integrin-deficient (β7^–/–^) mice at the time points indicated. Number of samples (n) has been demarcated under each time point wherein each symbol depicts one mouse. Colors, symbols, and numbers: WT mice (blue), β7^–/–^ mice (red), Females (circles), Males (squares), cages (dotted circles with corresponding number).

Hence, to decipher the function of β7 integrin-dependent immune cells in maintenance of the gut microbiota structure, fecal samples were collected from healthy, cohoused, wild-type and β7 integrin-deficient littermates of different age and gender from heterozygous breeding pairs (Figure 1). The study included the analysis of a total of 66 samples distributed into three age groups (6–7 w, 8–9 w, 10–11 w) for each genotype and gender.

**FIGURE 2:**
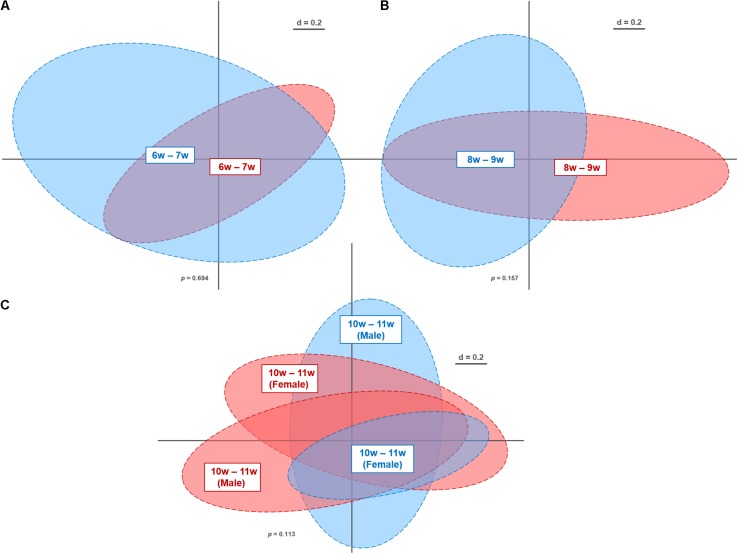
Non-metric multi-dimensional scaling plot of microbiota profiles from wild-type (WT) and β7 integrin-deficient (β7^–/–^) mice at different time points. Fecal samples from WT (*n* = 29; blue) and β7^–/–^ mice (*n* = 37; red) were analyzed by Illumina sequencing of 16S rRNA gene amplicons (V4 region; 233 bp). Similarities between microbiota profiles were calculated using generalized UniFrac distances in Rhea (beta-diversity). Individual time points and gender: 6–7 w **(A)**, 8–9 w **(B)**, 10–11 w **(C)** that were considered for calculations have been marked within their respective clusters.

Overall, we obtained 10,645,644 high-quality and chimera-checked sequences (155,432 ± 35,007 per sample) representing a total of 173 OTUs (159 ± 8 OTUs per sample). Sequencing depth was evaluated by means of rarefaction curves (Supplementary Figure S3). As apparent from the plateau observed in these curves, all samples passed the quality check and were therefore further analyzed. Differences in microbiota profiles between the genotypes were assessed at the different age windows by multidimensional scaling of phylogenetic distances (Figure 2). As evident from the NMDS plots, WT and β7 integrin-deficient mice displayed overall similar profiles at all of the analyzed time windows: 6–7 w (*p* = 0.694), 8–9 w (*p* = 0.157), 10–11 w (*p* = 0.252). Furthermore, we investigated gender-specific differences at time point 10–11 w, which included enough individual mice to allow robust statistical analysis. This reveals also no effect on microbiota profiles (*p* = 0.113) (Figure 2C). This is in contrast to studies attributing gender a role in host immunity and microbiome composition in other models (Haro et al., 2016; Elderman et al., 2018). Nonetheless, these studies were performed during immunological challenge or pathological state, while investigations in homeostasis are limited.

**FIGURE 3:**
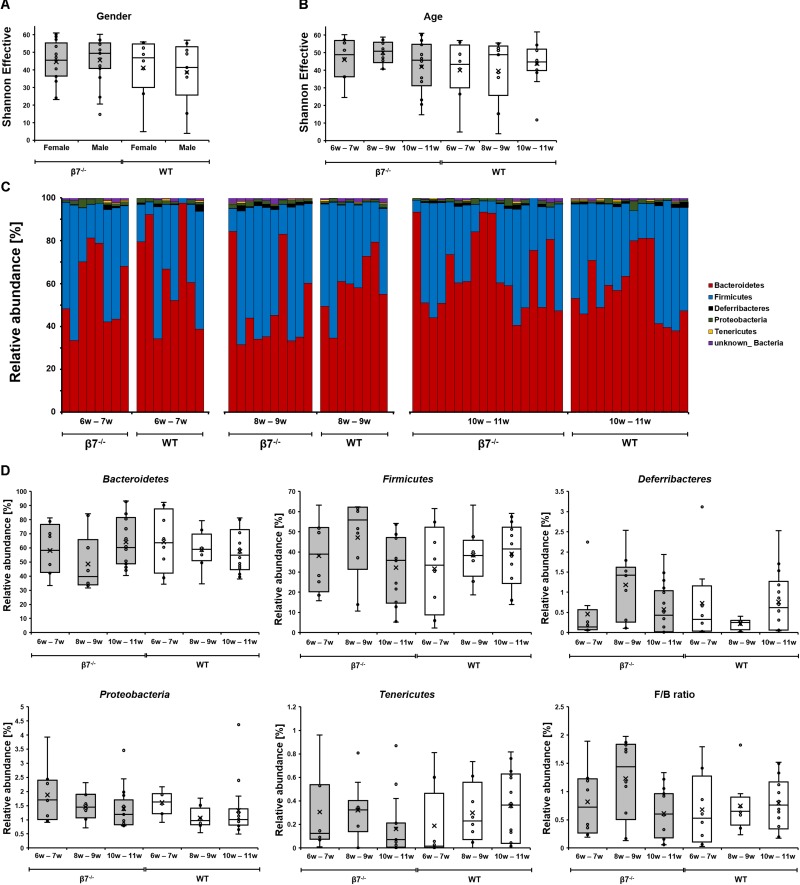
Diversity and composition of the different mouse groups. Box plots depicting the Shannon diversity in samples based on **(A)** Gender **(B)** Time (mouse age). The diversity of OTUs within a given sample (alpha-diversity) was calculated in Rhea. **(C,D)** Taxonomic binning of fecal microbiota of wild-type and β7 integrin-deficient mice at the phylum level. In all panels, WT mice are shown in white and β7^–/–^ mice in gray.

Some studies showed that the diversity of gut microbial communities can be altered by host age (O’Toole, 2012; Davenport et al., 2017; Dubois et al., 2017; Buford et al., 2018). The analysis of Shannon effective diversity revealed no difference between genotypes, genders, or the time points measured in our study (Figures 3A,B). Overall microbiota compositions in the fecal samples were stable between the two genotypes, e.g., relative abundances of the four most dominant phyla detected in our mice (*Bacteroidetes*, *Firmicutes*, *Deferribacteres* and *Proteobacteria*) did not show significant differences (Figure 3C). The *Firmicutes* to *Bacteroidetes* ratio (F/B), which has been examined in many studies in relation to inflammation and other pathological scenarios albeit with limited consensus in results (Sze and Schloss, 2016; Johnson et al., 2017), was also unchanged in β7 integrin-deficient mice. The relative abundances of bacterial groups at any taxonomic levels down to families revealed also no significant differences between the two genotypes. Hence, the compromised mucosal compartment of β7 integrin-deficient mice elicited no drastic effects on fecal microbiota structure in homeostasis.

**FIGURE 4:**
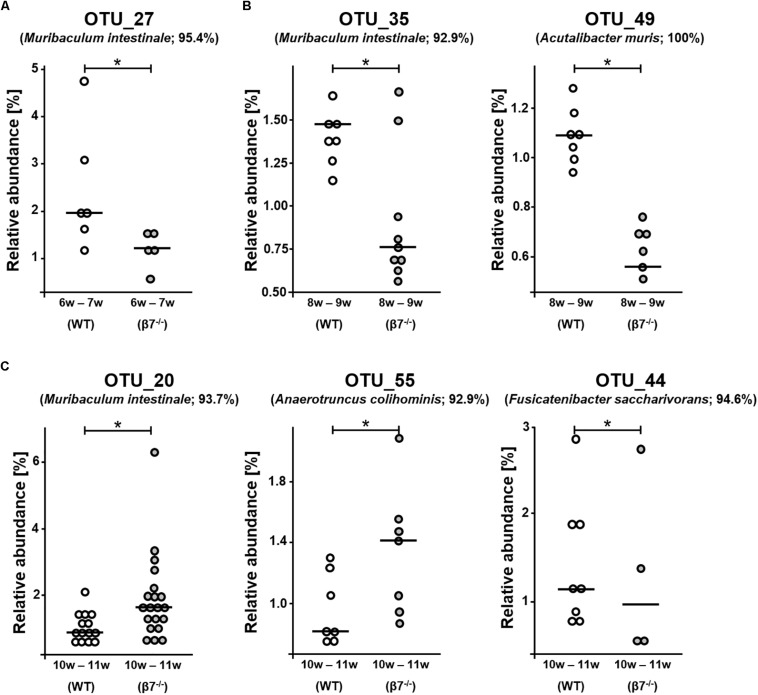
Analysis of single phylotypes. Relative abundances of Operational Taxonomic Units (OTUs) in wild-type (WT) and β7 integrin-deficient (β7^–/–^) mice at different time points **(A)** 6–7 w (OTU_27) **(B)** 8–9 w (OTU_35 and OTU_49) and **(C)** 10–11 w (OTU_20, OTU_55 and OTU_44). WT mice are shown in white and β7^–/–^ mice in gray. Significance was calculated by non-parametric ANOVA (Kruskal-Wallis Rank Sum Test), ^∗^*p* < 0.05.

We then analyzed differences at the level of single phylotypes. Out of the 173 detected OTUs, the occurrence of six molecular species was altered between the genotypes across all ages investigated (Figure 4). These were OTU_27: *Muribaculum* sp. 1 (95.4% sequence identity to *M. instestinale*) in 6–7 w samples; OTU_35: *Muribaculaceae* sp. 1 (92.9% to *M. instestinale*) and OTU_49: *Acutalibacter muris* (100%) in 8–9 w samples, and OTU_20: *Muribaculaceae* sp. 2 (93.7% to *M. instestinale*), OTU_55: *Ruminococcaceae* sp. 1 (92.9% to *Anaerotruncus colihominis*), and OTU_44: *Lachnospiraceae* sp. 1 (94.6% to *Fusicatenibacter saccharivorans*) in 10–11 w samples. Whilst statistically significant, these changes were rather singular and further suggest limited effects of β7 integrin on the fecal microbiota during homeostasis. Interestingly, three of the aforementioned OTUs are most closely related to the species *Muribaculum instestinale*, the reference species within the newly described family *Muribaculaceae*, formerly referred to as family *S24-7* within the phylum *Bacteroidetes* (Lagkouvardos et al., 2019). There have been several reports mentioning major shifts in *Muribaculaceae* spp. due to various diets, host conditions or colonization processes (Serino et al., 2012; Shen et al., 2016; Tropini et al., 2018). Another study by Osaka et al. (2017) mentioned overall decrement in the relative abundance of *Muribaculaceae*, *Lachnospiraceae* and *Ruminoclostridium* during inflammatory and healing phases in DSS Colitis in mice. Even though little is known about *Muribaculaceae* and changes affecting the relative abundances of the three unknown species identified in our study showed different directions (increase or decrease), family members were found to interact with innate and adaptive immune responses via Immunoglobulin A (IgA) coating (Bunker et al., 2015). Interestingly, *Muribaculaceae* was the only taxon found appreciably in the colonic IgA^–^ fraction and at >1% relative abundance in the duodenum but was also found enriched in the IgA^+^ fraction in both locations. Thus, nearly all colonic IgA^+^ taxa were also abundant in the small intestine, whereas most IgA^–^ taxa were abundant only in the colon (Bunker et al., 2015). On analyzing IgA coating in a model of CD4^+^ T-follicular helper cells hyper-sufficiency, IgA coating was clearly driven by exaggerated T cell-dependent responses against a single taxon, *Muribaculaceae* that did not require T-cells for IgA targeting (Bunker et al., 2015). Therefore, the changes in relative abundance of these bacteria could also reflect the reduced numbers of intestinal IgA secreting plasma cells and the defective specific T cell-dependent intestinal IgA responses observed in β7 integrin-deficient mice (Schippers et al., 2012). It would be very interesting to evaluate whether OTUs that show a different abundance in β7 integrin-deficient and wild-type mice show different IgA binding.

The primary focus of our study was to reveal GALT-dependent differences in the fecal microbiota compartment of WT and β7 integrin-deficient mice. Overall, we demonstrate that even though β7 integrin deficiency causes important changes in the gut mucosal immune system, it had little impact on the structure of dominant fecal bacterial communities in homeostasis, pointing at strong redundant compensatory mechanisms of the host for the maintenance of homeostatic balance. β7 integrin promotes IBD (Lamb et al., 2018), β7 integrin deficiency ameliorates experimental colitis in mice (Schippers et al., 2015), and IBD is accompanied by dysbiosis (Henson and Phalak, 2017). Therefore, one can speculate that the observed small differences in microbiota composition of WT and β7 integrin-deficient mice in homeostasis might accumulate to bigger changes in case of immunopathophysiology. Interestingly, preliminary results from our own ongoing study on these mice in an experimental model of Primary Sclerosing Cholangitis (PSC) [DDC (3,5-diethoxycarbonyl-1,4-dihydrocollidine)-induced PSC (Fickert et al., 2007)] show drastic differences in the microbiota diversity of feces from WT and β7 integrin-deficient mice (unpublished observations). In addition, the outcome of comparative microbiota profiling, even in homeostasis, might vary considerably when comparing microbiota profiles at intestinal regions other than feces, especially mucosal surfaces.

## Data Availability Statement

The datasets generated for this study can be found in the Sequence Read Archive – accession number PRJNA514431.

## Ethics Statement

The animal study was reviewed and approved by North Rhine-Westphalia (LANUV – Landesamt für Natur, Umwelt und Verbraucherschutz NRW) Recklinghausen, Germany (approval no. 81-02.04.2017.A429).

## Author Contributions

NW and AS designed the study. AB, JH, and SE performed the experiments and analyzed the data. JH performed the experiments and analyzed the ILC data. TH, TC, and OP helped with high throughput 16S rRNA gene sequence analysis and data interpretation. AB, TC, and AS wrote the manuscript. All authors reviewed and approved the final manuscript.

## Conflict of Interest

The authors declare that the research was conducted in the absence of any commercial or financial relationships that could be construed as a potential conflict of interest.

## Footnotes

1 https://www.arb-silva.de/
