# Supplementary material for: The Compromised Mucosal Immune System of β7 Integrin-Deficient Mice Has Only Minor Effects on the Fecal Microbiota in Homeostasis
Source: Front Microbiol. 2019 Oct 4;10:2284. doi: 10.3389/fmicb.2019.02284 (PMC6787405; doi:10.3389/fmicb.2019.02284)
Supplement: Supplementary file 1 [file Data_Sheet_1.PDF]

Supplementary Figure-1

A

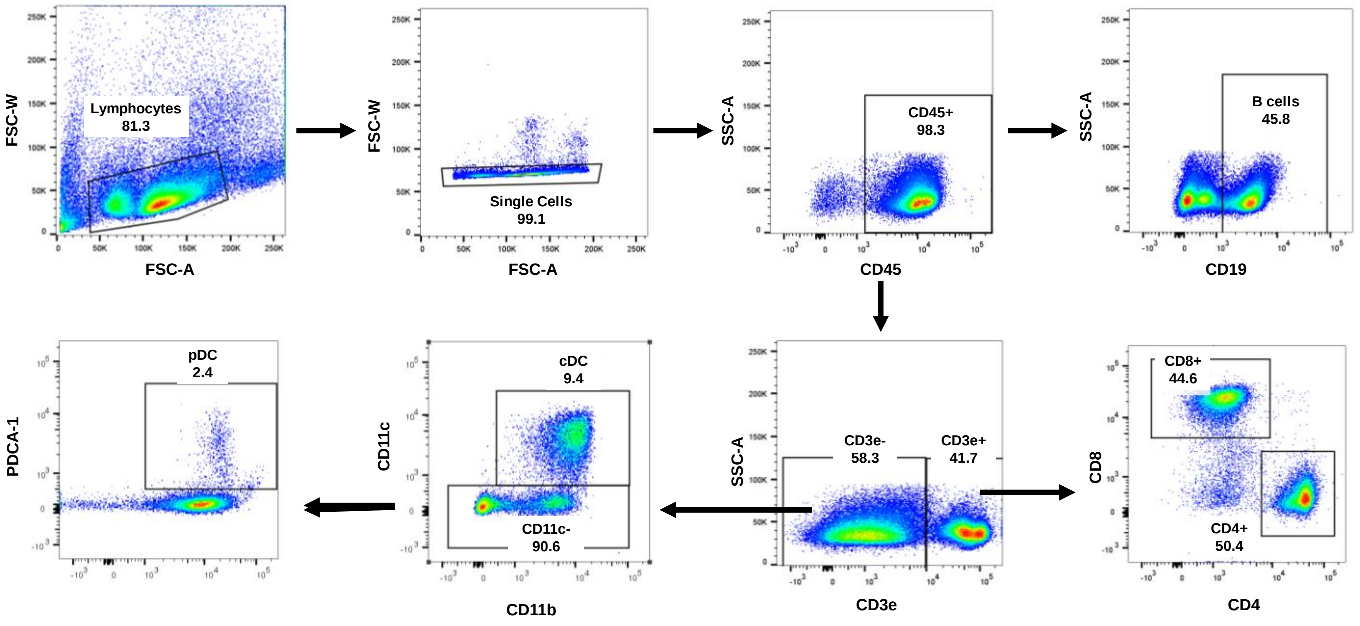

B

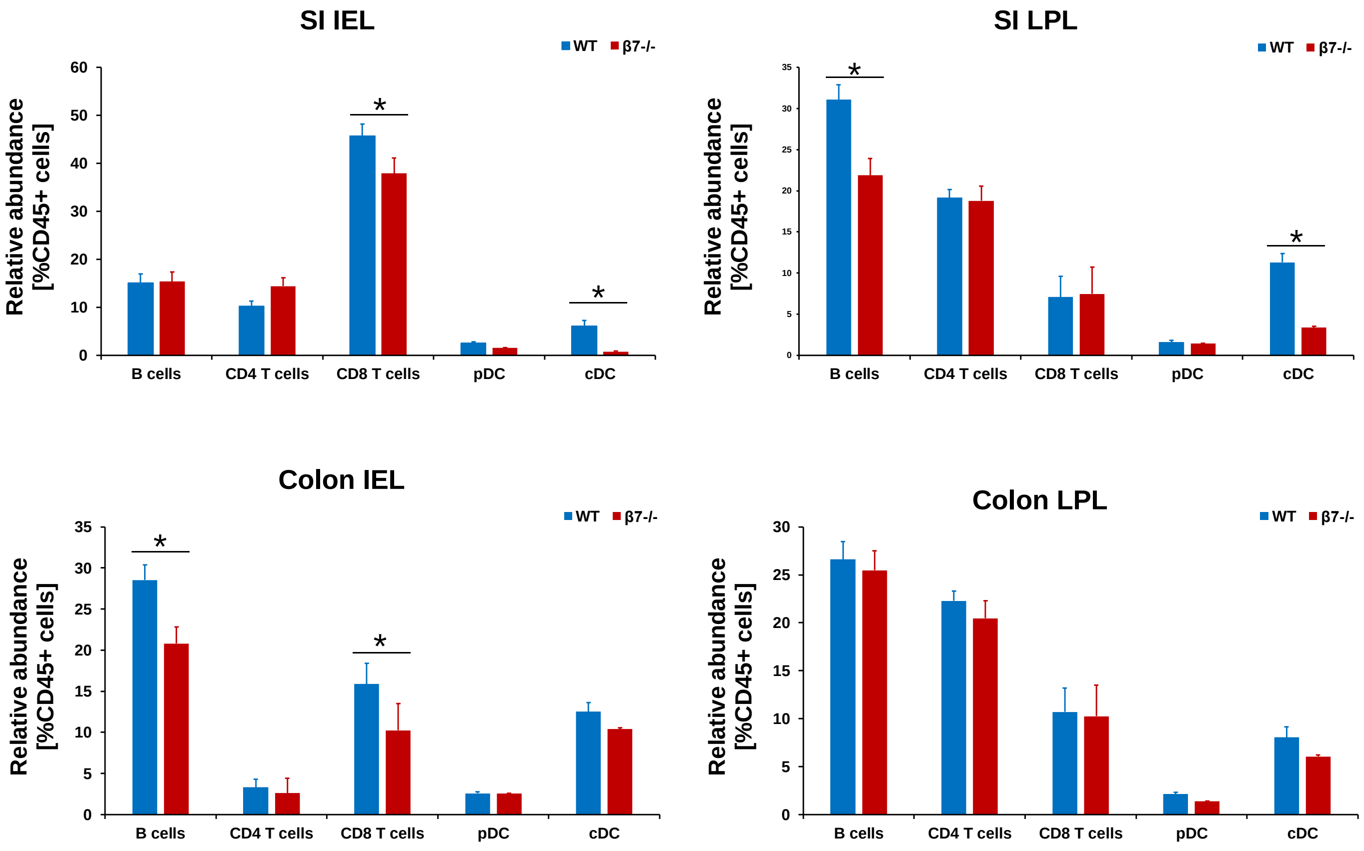

Figure S1: **Immune cell fractions of WT and β7 integrin deficient mice. (A)** Immune cell subset gating strategy by multiparameter flow cytometric analysis (WT small intestine IEL representative for all organ fractions). The analysis included B cells ($CD45^+CD19^+$), $CD4^+$ T cells ($CD45^+CD3^+CD4^+$), $CD8^+$ T cells ($CD45^+CD3^+CD8^+$), pDCs ($CD45^+CD3^-CD11b^-CD11c^+PDCA\text{-}1^+$) and cDC ($CD45^+CD3^-CD11b^+CD11c^+$). **(B)** IEL and LPL fractions of small intestine and colon were prepared and stained as mentioned before (Hadis et al., 2011). Age matched chow fed WT and $\beta7^{-/-}$ mice were used for the experiment. The cell numbers were counted by FACS and $\%CD45^+$ cells were plotted. One way ANOVA was used for all statistical analysis ($p < 0.05$, n = 8 – 10 per group).

Supplementary Figure-2

A

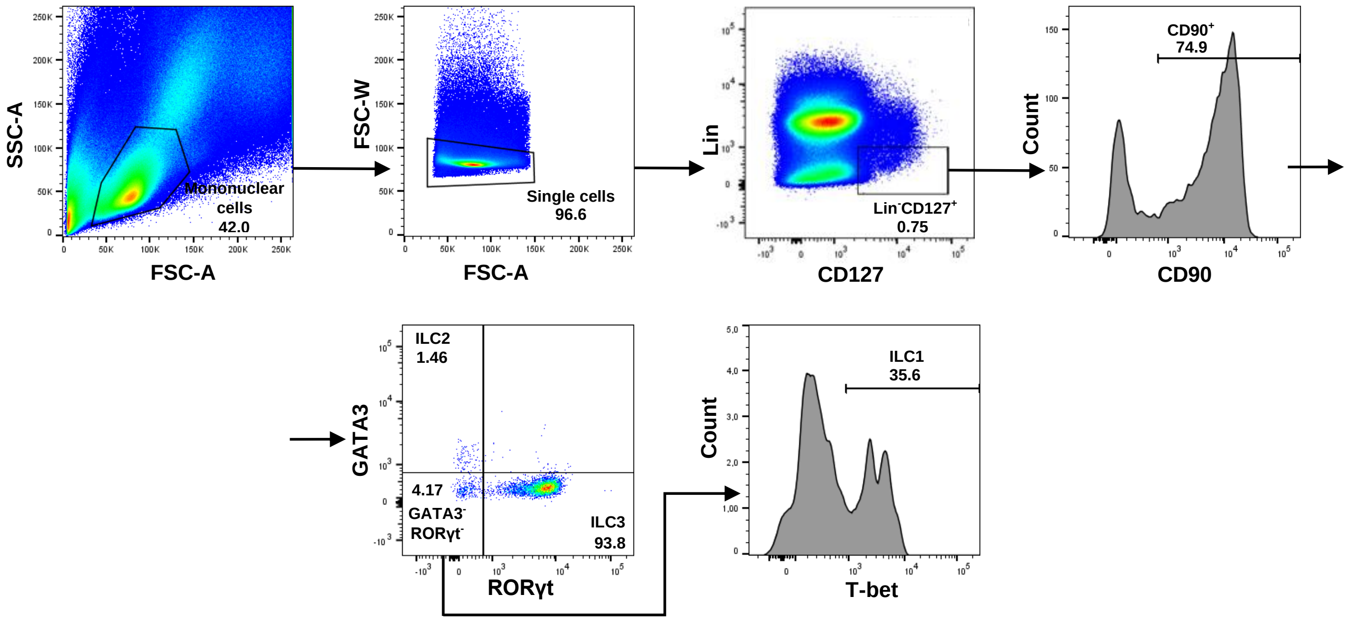

B

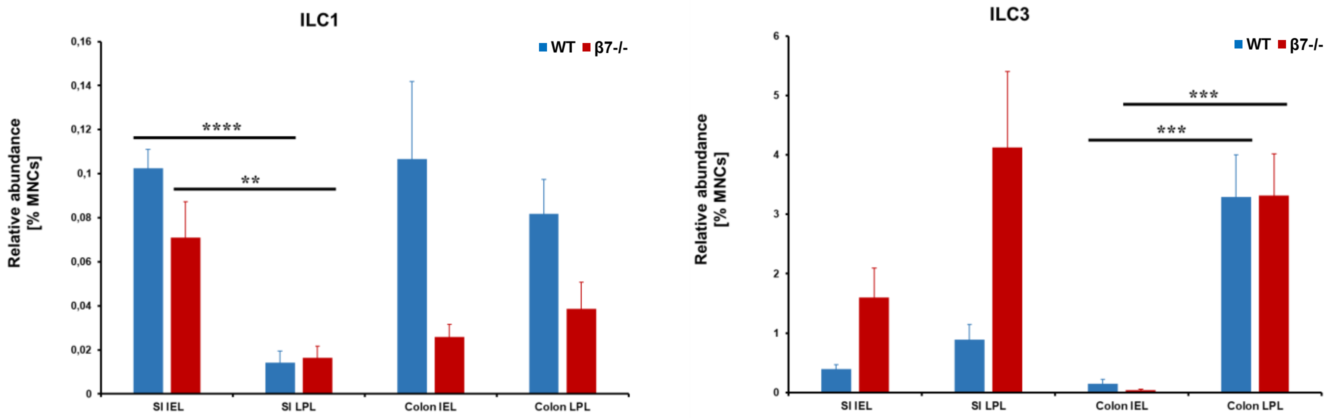

<u>Figure S2:</u> **Innate lymphoid cell fractions of WT and β7 integrin deficient mice. (A)** ILC subset gating strategy by multiparameter flow cytometric analysis (WT small intestine LPL representative for all organ fractions). ILC1 (Lin$^-$CD127$^+$CD90$^+$RORγt$^-$GATA3$^-$T-bet$^+$) and ILC3 (Lin$^-$CD127$^+$CD90$^+$RORγt$^+$GATA3$^-$). Lin (CD3, CD5, CD8, B220, CD11b, CD11c, NK1.1, Gr-1, F4/80, CD19 and TER-119). **(B)** Small intestine and colon IEL and LPL fractions of age matched chow fed WT and β7$^{-/-}$ mice were prepared as mentioned before (Hadis et al., 2011). The cell fractions as %MNCs were measured by flow cytometry and are shown as mean ±SEM. Two-tailed Mann-Whitney test was used for statistical analysis ($p < 0.05$, n = 9 – 15 per group, colon IEL n = 5 representing pools of 5 mice each).

Supplementary Figure-3

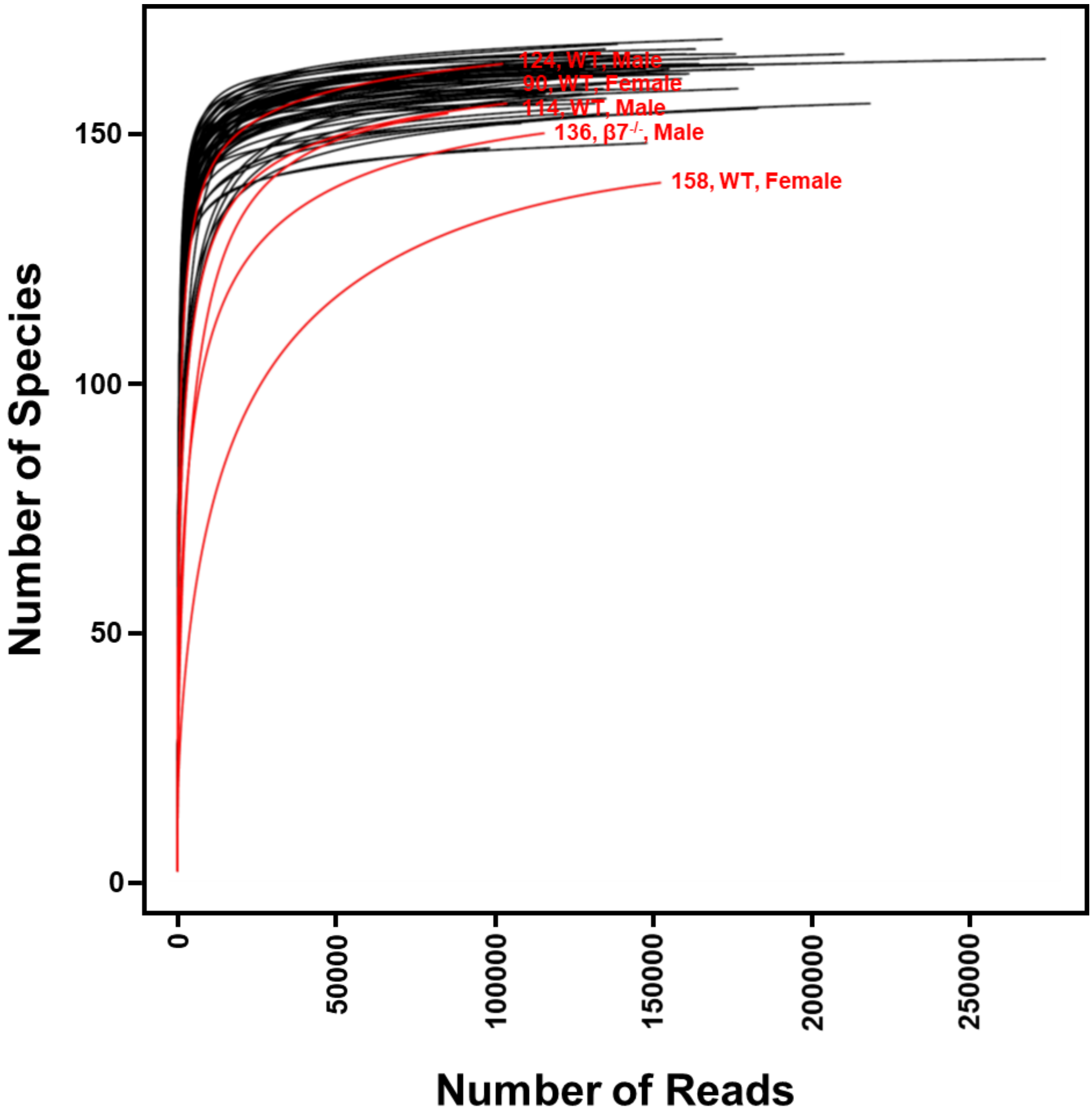

Figure S3: Rarefaction curves depicting sequencing depth. The number of observed species in each sample was plotted against the number of reads acquired, depicting a plateau region. The curve was generated using Rhea (Ilias Lagkouvardos, 2017) by standard normalized counts via simple division to their sample size and then multiplication by the size of the smaller sample. The five samples with the least sequencing reads have been depicted in red. Wild-type (WT), β7 integrin-deficient ($\beta7^{-/-}$).

References:

- Hadis, U., Wahl, B., Schulz, O., Hardtke-Wolenski, M., Schippers, A., Wagner, N., Müller, W., Sparwasser, T., Förster, R., and Pabst, O. (2011). Intestinal Tolerance Requires Gut Homing and Expansion of FoxP3+ Regulatory T Cells in the Lamina Propria. *Immunity* 34**,** 237-246.
- Ilias Lagkouvardos, S.F., Neeraj Kumar, Thomas Clavel (2017). Rhea: a transparent and modular R pipeline for microbial profiling based on 16S rRNA gene amplicons. *PeerJ* 5.
